# Adolescent deliveries in rural Cameroon: an 8-year trend, prevalence and adverse maternofoetal outcomes

**DOI:** 10.1186/s12978-017-0382-6

**Published:** 2017-09-29

**Authors:** Valirie Ndip Agbor, Clarence Mvalo Mbanga, Tsi Njim

**Affiliations:** 1 Ibal Sub-divisional Hospital, Oku, Northwest Region Cameroon; 2 Mankon Sub-divisional Hospital, Bamenda, Northwest Region Cameroon; 3 Centre for Tropical Medicine and Global Health, Nuffield Department of Medicine, University of Oxford, Oxfordshire, UK; 4 Health and Human Development Research Group (2HD), Douala, Cameroon

**Keywords:** Adolescent deliveries, Adverse maternal and foetal outcomes, Rural Cameroon

## Abstract

**Background:**

Adolescent deliveries remain a global public health concern especially in low- and middle-income countries where 95% of these deliveries occur. In Cameroon, adolescent pregnancies have a high disease burden due to their association with adverse pregnancy outcomes. We sought to evaluate the prevalence, trend and adverse maternofoetal outcomes of adolescent deliveries in a rural community in Cameroon.

**Method:**

We carried out a retrospective register analysis of 1803 singleton deliveries in two health facilities located in the Oku sub-division over an 8-year period (2009 to 2016). We excluded: records without maternal age, babies born before arrival, birthweights less than 1000 g, multiple deliveries and deliveries before 28 weeks gestation. Data analysis was done using Epi info 7.0.8.3. The Fisher’s exact test was used to compare categorical variables, while the chi-square test for trends was used to determine time trends. *P*-values below 5% were considered statistically significant.

**Results:**

The 8-year prevalence of adolescent deliveries was 20.4% (95% CI = 18.6–22.4) with a significant, downward trend between 2009 and 2016 (P trend = 0.05). Second-fourth degree perineal tears were more likely to complicate adolescent (Age < 20 years) deliveries compared with their adult (Age ≥ 20 years) counterparts (OR = 2.9; 95% CI = 1.8–4.7; *p* < 0.001). Also, babies born to adolescent mothers were more likely to have a low birthweight (OR = 1.7; 95% CI = 1.1–2.6; *p* = 0.009) and be asphyxiated at the fifth minute of life (OR = 3.2; 95% CI = 1.9–5.5; *p* < 0.001). Over an eight-year period, the downward trend in the prevalence of adolescent deliveries was associated with a significant decrease in the trend of neonatal asphyxia at the fifth minute. Married adolescents and their babies were as likely to develop the complications of adolescent delivery such as second-fourth degree perineal tears (OR = 0.8; 95% CI = 0.4–1.6; *p* = 0.456), low birthweight (OR = 2.1; 95% CI = 0.9–4.7; *p* = 0.070) and fifth minute neonatal asphyxia (OR = 0.9; 95% CI = 0.4–2.0; *p* = 0.832) as single adolescents and their babies.

**Conclusion:**

The prevalence of adolescent deliveries in this rural community is high with one of every five babies born to an adolescent mother. Despite the downward trend indicating a decrease in adolescent deliveries, our study demonstrates the need to reinforce and effectively apply existing government-based public health programme to target key indicators of adolescent pregnancy in Cameroon.

## Plain English summary

Pregnancy and delivery in adolescence is a major health problem worldwide, especially in low- and middle-income countries where over 95% of these deliveries occur. Like other low-income countries, the situation in Cameroon is marked by a high prevalence of adolescent pregnancies, and poor health outcomes for both the mother and her unborn baby. Most studies on adolescent deliveries in Cameroon have been conducted either in urban or semi-urban areas. To the best of our knowledge, this is the first study conducted in a rural area of the country to evaluate the prevalence and the adverse maternal and foetal outcomes of adolescent deliveries over an 8 year period.

We reviewed the delivery registers containing information on deliveries conducted within an 8-year period (2009–2016) in two health facilities in a rural sub-division in Cameroon. After excluding records without maternal age, babies born before arrival to the health facilities, birthweights less than 1000 g, multiple deliveries and deliveries before 28 weeks gestation; 1803 singleton delivery records were retained for this study.

Adolescent deliveries represented 20.4% of the 1803 deliveries included in this study. Also, the prevalence of adolescent deliveries significantly decreased over the 8-year study period. Interestingly, a similar decrease in birth asphyxia at the fifth minute of life was also noted. Moreover, compared with adult deliveries, adolescent deliveries were more likely to be complicated by a second-fourth degree perineal tear and these adolescent mothers were more likely to have low birthweight babies, and babies asphyxiated at the fifth minute of life. Finally, married adolescents and their babies were as likely to develop the complications of adolescent delivery such as second-fourth degree perineal tears, low birthweight and birth asphyxia at the fifth minute of life as single adolescents and their babies.

In conclusion, our study depicts a high prevalence of adolescent deliveries in this rural sub-division. In fact, it shows that one in every five babies is born to an adolescent mother. Even though, this community is experiencing a downward trend in the prevalence of adolescent deliveries, there is a dire need not only to reinforce the existing government-based public health policies to target the key indicators of adolescent pregnancy in Cameroon, but also to effectively apply them.

## Background

Varying definitions have been attributed to adolescent pregnancy. Generally, adolescent or teenage pregnancy refers to pregnancy occurring in a girl aged 13 to 19 years. Adolescent pregnancy is a major public health concern worldwide, especially in sub-Saharan Africa. Globally, 11% of all births result from adolescent pregnancy, with 95% of these births occurring in low- and middle-income countries [1]. In fact, the complications of adolescent pregnancy and childbirth is the second cause of death among girls aged 15–19 years worldwide [1], and this is 2.5 times higher in girls below 15 years of age [1]. Factors such as: customs and traditions that encourage early marriages, poor knowledge on reproductive sexual health, wrong contraceptive use (as over half of unmarried adolescents do not use effective contraception) have been attributed to adolescent pregnancy [1–4]. Delivery in adolescence is associated with higher risks of hypertensive disorders, caesarean delivery, low birthweight (LBW), neonatal asphyxia, prematurity and stillbirth [1, 3, 5–7].

The situation of adolescent pregnancy in Cameroon, like other sub-Sahara African countries, needs to be addressed. Adolescent girls constitute 23.2% (5.4 million) of the total Cameroonian population, 44.5% of whom reside in rural areas [4]. About 84% of the married, and half of unmarried, sexually active adolescent girls in Cameroon are not on any contraceptive method [4]. By 2011, 25% of adolescent girls in Cameroon had already begun their reproductive life; 21% of whom had at least a child and 4% were pregnant for their first time. In addition, the proportion of adolescent girls who had already begun their reproductive life was remarkably elevated in the rural (34%) compared to urban areas (18%) [4]. Contrasting prevalences of adolescent pregnancy have been reported throughout the national territory ranging from 2.8–26.5% [5, 6, 8–10]; with a national prevalence of 14.2% [10]. The burden of adolescent deliveries is likely to be higher in rural communities where education, especially education on reproductive sexual health is poorer. Most of the studies on adolescent pregnancy in Cameroon were mostly focused on urban and sub-urban areas. Also, there is the common misconception that married adolescents are more equipped to deal with the burden of pregnancy than their single counterparts. However, studies have reported no difference in the complications of adolescent deliveries between married and single adolescents [6]. To add to the body of knowledge on this subject, we carried out this study to evaluate the trend in the prevalence and adverse maternofoetal outcomes of adolescent deliveries; and to determine if married adolescents were precluded from having adverse outcomes when compared to single adolescents in two health facilities in rural Cameroon.

## Methods

### Study design, duration and setting

We conducted a retrospective – register analysis of delivery records during a period of 8 years, from January 1st, 2009 to December 31st, 2016, in two health facilities in the Oku Health District (OHD): Oku district hospital and Kevu primary health centre. These facilities conduct, in roughly equal proportions, about 40% of an average of 900 deliveries per month reported in this Health District. The OHD is in the Oku sub-division of the Bui – division, Northwest Region Cameroon. During the study period, the OHD was managed by three medical practitioners. The Oku district hospital was managed by a single doctor, while the Kevu primary health centre was managed by a nurse with no training in midwifery. Consequently, all caesarean sections were done at the district hospital. Referral in the health district is usually from the primary health care centres to the district hospital. This referral pattern is usually ineffective due to difficulties in accessing the main district hospital. The OHD has an estimated population of 93, 000 inhabitants, and majority of whom live below the poverty limit of one dollar per day.

### Data collection

All singleton deliveries recorded within the study period were included. Records: without maternal age (82% of excluded records), babies born before arrival (3.1% of excluded records), birthweights less than 1000 g (4.3% of excluded records), multiple deliveries (4.5% of excluded records) and deliveries before 28 weeks gestation (6.2% of excluded records) were excluded from our study.

We collected data on the sociodemographic characteristics of the study population (age, marital status), clinical characteristics (gravidity, parity, gestational age, human immunodeficiency virus [HIV] status and sex of the neonates), maternal outcome (mode of delivery, postpartum haemorrhage and second-fourth degree perineal tear) and foetal outcome (birthweight, fifth minute Apgar score and term of the pregnancy which was determined from the gestational age). Caesarean deliveries were only recorded as from the year 2014.

### Statistical analysis

All data were entered and analysed using Epi info 7.0.8.3 software after verifications for errors. Before statistical analysis, variables were categorised as shown in Table 1. Quantitative variables were presented as means with their corresponding standard deviation (SD) while categorical variables were presented as frequencies and percentages. A Fisher’s exact test was used to compare categorical variables. The Odd’s ratio (OR) with corresponding 95% confidence interval (CI) were used to estimate the degree of association that could exist between categorical variables. The Chi-square test for trends was used to determine time trends. *P*-values below 5% were considered statistically significant.

**Table 1:** Definition of operational variables

Age	Adolescent (<20 years) versus adult deliveries (≥20 years)
Adolescents	Early adolescence (10–16 years) versus late adolescence (17–19 years) [6].
Gravidity	1. Primigravida (Women at their first pregnancy) 2. Multigravida (2–4 pregnancies) 3. Grand multigravida (>4 pregnancies)
Parity	1. Primiparous (Women at their first delivery) 2. Multiparous (2–4 deliveries) 3. Grand multiparous (>4 deliveries)
Gestational age	1. Preterm delivery: Delivery from 28 to 36 weeks of gestation 2. Term delivery: Delivery from 37 to 42 weeks of gestation 3. Post-term delivery: Delivery above 42 weeks of gestation
Apgar score at fifth minute	Neonatal asphyxia. Yes (<7) versus No (≥7)
Low birthweight	Babies born with a weight ≤ 2600 g [28].
High birthweight	Babies born with a weight ≥ 3850 [29].

## Results

We included 1803 of the 2343 deliveries recorded within our defined study period, giving a response rate of 77%. The proportion of included delivery records were roughly the same for both facilities (District Hospital = 905 [50.2%] versus Health centre = 898 [49.8%]). The ages of the 1803 records analysed ranged from 14 to 49 years, with a mean age of 26.0 ± 6.5 years. Only two cases of delivery in girls below 15 years of age were noted. Adolescent deliveries constituted 368 of the 1803 deliveries, representing a prevalence of 20.4% (95% CI = 18.6–22.4). Of the 368 adolescents 40.5% were married while 22% were multiparous. Eleven (3.0%) of the 368 adolescents were positive for HIV (Table 2). The rate of caesarean deliveries was at 0.9%.

**Table 2:** Characteristics of the study population

Variable	Adolescent, N (%) = 368 (20.4)		Adult, N (%) = 1435 (79.6)	Total N (%) = 1803 (100)
	Early adolescent, N (%) = 57 (15.5%)	Late adolescent, N (%) = 311 (84.5)		
Marital status				
Single	47 (82.5)	171 (55.0)	220 (15.4)	439 (24.5)
Married	10 (17.5)	140 (45.0)	1207 (84.6)	1356 (75.5)
Gravidity (*n* = 1760)				
Primigravida	52 (91.2)	230 (75.2)	153 (11.0)	434 (24.7)
Multigravida	5 (8.8)	76 (24.8)	769 (55.0)	850 (48.3)
Grand multigravida	0 (0.0)	0 (0.0)	478 (34.0)	478 (27.0)
Parity (*n* = 1761)				
Primiparous	53 (93.0)	232 (75.8)	162 (11.6)	447 (25.4)
Multiparous	4 (7.0)	74 (24.2)	764 (54.6)	842 (47.8)
Grand multiparous	0 (0.0)	0 (0.0)	472 (33.8)	472 (26.8)
Gestational age (*n* = 1647)				
Term	28 (54.9)	181 (64.2)	820 (62.4)	1029 (62.5)
Preterm	21 (41.2)	81 (28.7)	436 (33.2)	538 (32.7)
Post term	2 (3.9)	20 (7.1)	58 (4.4)	80 (4.8)
Mode of delivery (*n* = 1802)				
Vaginal	57 (100.0)	308 (99.4)	1421 (99.0)	1786 (99.1)
Caesarean	0 (0.0)	2 (0.6)	14 (1.0)	16 (0.9)
Maternal HIV status				
Positive	1 (1.7)	10 (3.2)	70 (4.9)	81 (4.5)
Negative	56 (98.3)	301 (96.8)	1362 (95.1)	1719 (95.5)
Gender of infant				
Male	36 (45.6)	157 (50.5)	729 (61.0)	911 (51.1)
Female	31 (54.4)	154 (49.5)	701 (49.0)	886 (48.9)

*HIV* human immunodeficiency virus, *N* frequency

Adolescent deliveries were significantly associated with: second – fourth degree perineal tears (OR = 2.9; 95% CI = 1.8–4.7; *p* < 0.001), LBW (OR = 1.7; 95% CI = 1.1–2.6; *p* = 0.009) and neonatal asphyxia at the fifth minute of life (OR = 3.2; 95% CI = 1.9–5.5; *p* < 0.001), compared with adult deliveries (Tables 3 and 4). Seven cases of postpartum haemorrhage were recorded in this study, all of which resulted from adult deliveries. Married adolescents were as likely to have complications from deliveries as single adolescents (Table 5).

**Table 3:** Maternal outcomes of adolescent deliveries

Outcome	Adolescent (Age < 20 years)	Adult (Age ≥ 20 years)	OR (95% CI)	*p* – value
Postpartum haemorrhage				
Yes, n (%)	0 (0.0)	7 (0.5)	–	*–*
No, n (%)	368 (100.0)	1796 (99.5)		
Caesarean delivery				
Yes, n (%)	2 (0.5)	14 (1.0)	0.6 (0.1–2.6)	0.337
No, n (%)	365 (99.5)	1421 (99.0)		
Second - fourth degree perineal tear				
Yes n (%)	32 (8.7)	45 (3.1)	2.9 (1.8–4.7)	**<0.001**
No, n (%)	336 (91.3)	1390 (96.7)		

*OR* odd’s ratio, *CI* confidence interval, *n* frequency

**Table 4:** Foetal outcomes of adolescent deliveries

Outcome	Adolescent (Age < 20 years)	Adult (Age ≥ 20 years)	OR (95% CI)	*p* – value
Low birthweight				
Yes, n (%)	35 (9.8)	84 (5.9)	1.7 (1.1–2.6)	**0.009**
No, n (%)	324 (90.2)	1329 (94.1)		
High birthweight				
Yes, n (%)	10 (2.8)	94 (6.7)	0.4 (0.2–0.9)	**0.004**
No, n (%)	349 (97.2)	1320 (93.3)		
Neonatal asphyxia (5th min Apgar)				
Yes, n (%)	24 (6.7)	31 (2.2)	3.2 (1.9–5.5)	**<0.001**
No, n (%)	335 (93.3)	1379 (97.8)		
Stillbirth				
Yes, n (%)	7 (1.9)	18 (1.3)	1.5 (0.6–3.7)	0.233
No, n (%)	360 (98.1)	1417 (98.7)		
Preterm deliveries				
Yes, n (%)	102 (30.6)	436 (33.2)	0.9 (0.7–1.2)	0.396
No, n (%)	231 (69.4)	878 (66.8)		
Post-term deliveries				
Yes, n (%)	22 (6.6)	58 (64.4)	1.5 (0.9–2.5)	0.067
No, n (%)	311 (93.4)	1259 (95.6)		

*OR* odd’s ratio, *CI* confidence interval, *n* frequency

**Table 5:** Comparison of maternal and foetal outcomes between single and married adolescents

Outcome	Single adolescent	Married adolescent	OR (95% CI)	*p*-value
Second – fourth degree perineal tear				
Yes, n (%)	17 (7.8)	15 (10.1)	0.8 (0.4–1.6)	0.456
No, n (%)	202 (92.2)	134 (89.9)		
Low birthweight				
Yes, n (%)	26 (12.2)	9 (6.2)	2.1 (0.9–4.7)	0.070
No, n (%)	187 (87.8)	137 (93.8)		
Neonatal asphyxia at 5th minute of life				
Yes, n (%)	15 (7.0)	9 (6.2)	0.9 (0.4–2.0)	0.832
No, n (%)	198 (93.0)	137 (93.8)		

*OR* odd’s ratio, *CI* confidence interval, *n* frequency

**Fig. 1:**
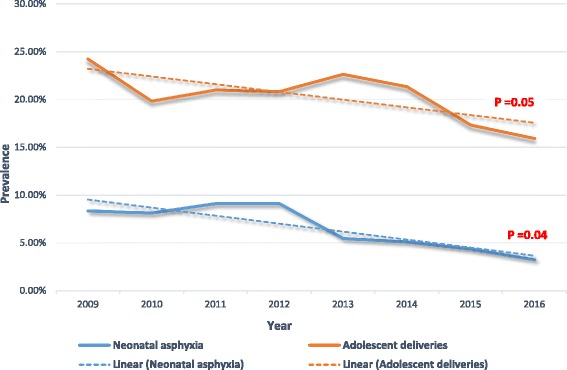
Trend in the prevalence of adolescent deliveries and neonatal asphyxia at the fifth minute in babies of adolescent mothers from the year 2009 to 2016

We noticed a significant downward trend in the annual prevalence of adolescent deliveries (from a prevalence of 24.2% [58/240] in the year 2009 to 15.9% [32/201] in 2016; P trend = 0.05) and neonatal asphyxia at fifth minute of life among babies of adolescent mothers (from a prevalence of 8.3% [5/60] in the year 2009 to 3.2% [1/31] in 2016; P trend = 0.04) over the 8-year period, Fig. 1. However, we observed no significant trend in the prevalence of the following adverse outcomes of adolescent deliveries: LBW (P trend = 0.61) and second – fourth perineal tear (P trend = 0.35).

## Discussion

Our study demonstrates that adolescent deliveries represent one-fifth of the total deliveries in the Oku sub-division. Even though this prevalence falls within the range 2.8–26.5% [5, 6, 8–10] reported in Cameroon, it is much higher than most reports from studies conducted in urban [8, 10] and semi-urban [5, 6, 10] settings. In addition, this prevalence follows closely the high prevalence of adolescent deliveries of 26.5% recorded in 2005 in Maroua, Cameroon [10]; a city where early marriages are a part of the cultural values of the people [10]. Indeed, almost half of the adolescents in our study were married. Factors such as: encouragement of early marriages, poverty, lack of proper education on sexual and reproductive health, decrease awareness and wrong use of contraceptive methods, and low educational ambition have been attributed to high rates of adolescent pregnancies, and consequently, adolescent deliveries in low-income countries [11]. Also, there was no difference in delivery outcomes between married adolescents and single adolescents. This finding emphasises the need to address the sexual and reproductive health needs for unmarried adolescents, who usually get less health care support than their married counterparts. In this rural area, with a high prevalence of early marriages, our study showed that married adolescents were just as likely to have complications from deliveries as their single counterparts. These findings were similar to those of Njim et al. who reported a similar risk of adverse delivery outcomes between adolescents and their adult counterparts [6]. This supports the assertion that adolescents might be generally ill-equipped to deal with the burden of pregnancy.

In this study, second – fourth degree perineal tears were about three times more likely to complicate adolescent than adult deliveries. Similar findings have been reported across other developing countries [12, 13]. This predisposition has been attributed to a biological immaturity, particularly pelvic and perineal immaturity in adolescents [5, 8, 14]. In addition, nulliparity, a condition which is relatively common in adolescent than adult women, has been shown to increase the risk of potentially severe and devastating fourth degree perineal tears by seven folds [15, 16]. However, our finding contrasts with those of Fouelifack et al. [8] and Ngowa et al. [9]. During labour, in the study carried out by Fouelifack et al., an episiotomy was immediately done in participants when an increased likelihood of a perineal tear was anticipated in a participant [8]. More so, they found that the odds for an episiotomy to be done was twice greater among adolescents compared with their adult counterparts. This could explain why perineal tears were unlikely to complicate adolescent deliveries in their study. Perineal tears have been attributed to severe and stigmatising complications like obstetric fistulae [17].

The rate of caesarean deliveries in this sub-division was unacceptably low, compared with the recommended 5–15% caesarean section rate by World Health Organisation (WHO) [18]. This was probably due to the fact that caesarean sections were conducted only at the district hospital where the doctor was stationed, and that caesarean deliveries were only recorded as from the year 2014. Also, factors handicapping a smooth referral system in this district could account for such a low caesarean section rate. Indeed, the mountainous topography of this health district which results in longer referral time, and a relatively high cost of transportation greatly hinders a smooth referral system. Consequently, health facilities distant to the main district hospital, like the Kevu health centre, prefer referring cases requiring emergency caesarean section to nearby districts. Adolescent deliveries were not associated with an increase rate of caesarean deliveries in this study. This finding contrasts with those of many authors [19, 20]. However, our finding tied with that of Njim et al. [6] and Fouelifack et al. [21]*.*

Adolescent deliveries were significantly associated with LBW and neonatal asphyxia at the fifth minute of life. These findings corroborate with other reports from Africa [5, 6, 14, 16]. However, we found no significant association between adolescent pregnancy, stillbirth and preterm delivery reported by other authors [5, 6, 22, 23]. Furthermore, it has been stipulated that due to a deficient production of oxytocin secondary to immaturity of the hypothalamo-epiphyseal pathway, and uterine immaturity, adolescent pregnancies have a greater tendency to persist beyond term compared with adult pregnancies [21]. Nevertheless, our study, among others [6, 7, 10, 19], showed that adolescent pregnancies are unlikely to exceed the normal term.

Over an 8-year period (2009–2016), there was a remarkable downward trend in the prevalence of adolescent deliveries in this sub-division. Even though, the prevalence of adolescent deliveries remain unacceptably high (15.9% in 2016; Fig. 1). This downward trend in the prevalence corresponds with the decreasing prevalence of adolescent girls who have already commenced their reproductive life in Cameroon, as reported by the World bank and investigators of the Demographic and Health Survey (DHS), 2011 [4, 24]. Over the years, the Ministry of Public Health in Cameroon has developed policies to address sexual reproductive health such as: the National Population Policy in 1992, the “Maternal and Child Health Care and Family Planning Services Policy and Standards” in 1995, and the “Roadmap for Reduction of Maternal and Neonatal Mortality in Cameroon 2006 – 2015” [25]. It is noteworthy that none of these policies specifically address adolescent pregnancy and its associated burden [25]. The “Roadmap for Reduction of Maternal and Neonatal Mortality in Cameroon 2006–2015” was supported by the bodies like the WHO, UNICEF and United Nation Development Programme (UNDP), and had as main goal to ameliorate delivery of reproductive health care services, procuring of qualified health personnel, while enhancing capacity building and family planning services for Cameroon’s communities [26]. The “Roadmap” has been the main pillar to meet the reproductive and sexual health of the Cameroonian population. To prevent adolescent pregnancies, the “Roadmap” in association with the Department in-charge of Health Promotion programmed education of youngsters at community levels on how to prevent adolescent pregnancies. However, the details on how these youngsters were to be educated was not detailed in the “Roadmap”. The lack of detailed specific objectives on how to prevent adolescent pregnancy may have attributed to Cameroon not attaining the Millennium Development Goal (MDG) five. Amelioration and effective application of existing government policies to curb the prevalence of adolescent pregnancy and its related burden may go a long way in attaining the Sustainable Development Goal (SDG) three set for the year 2030 [27]. Indeed, the downward trend in the prevalence of adolescent deliveries was associated with a significant, downward trend in the prevalence of neonatal asphyxia at the fifth minute of life of babies born to adolescent mothers. This depicts the impact of public health interventions on the adverse outcome of adolescent deliveries.

## Study limitation

Since this was a retrospective study, we had no influence on the quality of data entered into the delivery registers. Also, this study estimated the prevalence of adolescent deliveries. This might not represent the true prevalence of adolescent deliveries as we did not include abortions and could not estimate if the proportions of adolescents using health facilities were similar to those of adults. However, with the large sample size, the results reflect to a certain degree the situation of adolescent deliveries in rural areas in Bui division.

## Conclusion

Our study reveals a high prevalence of adolescent deliveries in this rural community in Cameroon. Adolescent mothers are more likely to sustain second - fourth degree perineal tears during labour and their babies have a higher tendency of being asphyxiated and/or having LBW. Married adolescents were not precluded from having adverse maternofoetal outcomes when compared with single adolescents. The prevalence of adolescent deliveries has experienced a remarkable downward trend, with a corresponding decreasing trend in the prevalence of neonatal asphyxia at the fifth minute of life in babies born to adolescent mothers, over an 8-year period. Despite this downward trend, the prevalence of adolescent deliveries in this rural community remains high. We recommend not just a reinforcement, but also an effective application of the existing government-based policies to specifically target key indicators of adolescent pregnancy, in order to curb its related complications especially in the rural areas. Also, studies to evaluate qualitative factors such as cultural changes, religious influence and increased service delivery that could explain the observed trend in the prevalence of adolescent deliveries are warranted in this sub-division.

## Abbreviations

CI: Confidence interval
HIV: Human immunodeficiency virus
LBW: Low birthweight
NGO: Non-governmental Organisation
OR: Odd’s ratio
